# Supplementary material for: Immunotherapy Combined with Chemotherapy in the First-Line Treatment of Advanced Gastric Cancer: Systematic Review and Bayesian Network Meta-Analysis Based on Specific PD-L1 CPS
Source: Curr Oncol. 2025 Feb 16;32(2):112. doi: 10.3390/curroncol32020112 (PMC11854702; doi:10.3390/curroncol32020112)
Supplement: Supplementary file 1 [file curroncol-32-00112-s001.zip › curroncol-3415904-supplementary.pdf]

Immunotherapy combined with chemotherapy in the first-line treatment of advanced gastric cancer: systematic review and Bayesian network meta-analysis based on specific PD-L1 CPS

Wenwei Zhang, Kaibo Guo and Song Zheng

Supplementary materials

Table of Contents

		Page
Table S1	Checklist of the PRISMA extension for network meta-analysis	2-5
Table S2	Literature search criteria	5
Table S3	Bayesian ranking results of network meta-analysis	5-7
Table S4	Probability of occurrence of a specific adverse event	7-8
Table S5	Comparisons of the fit of consistency and inconsistency models	8-9
Table S6	Bayesian ranking results of the sensitivity analysis	9-10
Figure S1	Convergence of the three chains established by inspection of the history feature	10-14
Figure S2	Summary of results from assessment of studies using the Cochrane risk of bias tool	14
Figure S3	The probability of a specific adverse event	15
Figure S4	Forest plots depicting results of head-to-head comparisons based on frequentist pairwise meta-analyses	15
Figure S5	Network diagrams for the first sensitivity analysis	16
Figure S6	Pooled estimates of the first sensitivity analysis	17-18
Figure S7	Bayesian ranking profiles for the first sensitivity analysis	19-20
Figure S8	Leave-one-out sensitivity analyses	20
Figure S9	Network diagrams for the second sensitivity analysis	21
Figure S10	Pooled estimates of the second sensitivity analysis	21-23
Figure S11	Bayesian ranking profiles for the second sensitivity analysis	24

Table S1. Checklist of the PRISMA extension for network meta-analysis.

**Table S1. PRISMA NMA Checklist of Items to Include When Reporting a Systematic Review Involving a Network Meta-analysis**			
Section/Topic	Item #	Checklist Item	Reported on Page #
**TITLE**			
Title	1	Identify the report as a systematic review incorporating a network meta-analysis (or related form of meta analysis).	1
**ABSTRACT**			
Structured summary	2	Provide a structured summary including, as applicable:<br>• Background: main objectives;<br>• Methods: data sources; study eligibility criteria, participants, and interventions; study appraisal and synthesis methods, such as network meta analysis.<br>• Results: number of studies and participants identified; summary estimates with corresponding confidence/credible intervals; treatment rankings may also be discussed. Authors may choose to summarize pairwise comparisons against a chosen treatment included in their analyses for brevity.<br>• Discussion/Conclusions: limitations; conclusions and implications of findings.<br>• Other: primary source of funding; systematic review registration number with registry name.	1-2
**INTRODUCTION**			
Rationale	3	Describe the rationale for the review in the context of what is already known, including mention of why a network meta-analysis has been conducted.	2-4
Objectives	4	Provide an explicit statement of questions being addressed, with reference to participants, interventions, comparisons, outcomes, and study design (PICOS).	4
**METHODS**			
Protocol and registration	5	Indicate if a review protocol exists, if and where it can be accessed (e.g., Web address), and, if available, provide registration information including registration number.	4-5
Eligibility criteria	6	Specify study characteristics (e.g., PICOS, length of follow-up) and report characteristics (e.g., years considered, language, publication status) used as criteria for eligibility, giving rationale. Clearly describe eligible treatments included in the treatment network, and note whether any have been clustered or merged into the same node (with justification).	5-6
Information sources	7	Describe all information sources (e.g., databases with dates of coverage, contact with study authors to identify additional studies) in the search and date last searched.	5
Search	8	Present full electronic search strategy for at least one database, including any limits used, such that it could be repeated.	Table S2

Study selection	9	State the process for selecting studies (i.e., screening, eligibility, included in systematic review, and, if applicable, included in the meta-analysis).	5-6
Data collection process	10	Describe method of data extraction from reports (e.g., piloted forms, independently, in duplicate) and any processes for obtaining and confirming data from investigators.	6
Data items	11	List and define all variables for which data were sought (e.g., PICOS, funding sources) and any assumptions and simplifications made.	6-7
Geometry of the network	S1	Describe methods used to explore the geometry of the treatment network under study and potential biases related to it. This should include how the evidence base has been graphically summarized for presentation, and what characteristics were compiled and used to describe the evidence base to readers.	6-7
Risk of bias in individual studies	12	Describe methods used for assessing risk of bias of individual studies (including specification of whether this was done at the study or outcome level), and how this information is to be used in any data synthesis.	7
Summary measures	13	State the principal summary measures (e.g., risk ratio, difference in means). Also describe the use of additional summary measures assessed, such as treatment rankings and surface under the cumulative ranking curve (SUCRA) values, as well as modified approaches used to present summary findings from meta-analyses.	7
Synthesis of results	14	Describe the methods of handling data and combining results of studies for each network meta-analysis. This should include, but not be limited to:<br>• Handling of multi-arm trials;<br>• Selection of variance structure;<br>• Selection of prior distributions in Bayesian analyses; and<br>• Assessment of model fit.	7-8
Assessment of Inconsistency	S2	Describe the statistical methods used to evaluate the agreement of direct and indirect evidence in the treatment network(s) studied. Describe efforts taken to address its presence when found.	7
Risk of bias across studies	15	Specify any assessment of risk of bias that may affect the cumulative evidence (e.g., publication bias, selective reporting within studies).	7
Additional analyses	16	Describe methods of additional analyses, if done, indicating which were pre specified. This may include, but not be limited to the following:<br>• Sensitivity or subgroup analyses;<br>• Meta-regression analyses;<br>• Alternative formulations of the treatment network; and<br>• Use of alternative prior distributions for Bayesian analyses (if applicable).	7-8
**RESULTS**			
Study selection	17	Give numbers of studies screened, assessed for eligibility, and included in the review, with reasons for exclusions at each stage, ideally with a flow diagram.	8

Presentation of network structure	S3	Provide a network graph of the included studies to enable visualization of the geometry of the treatment network.	8
Summary of network geometry	S4	Provide a network graph of the included studies to enable visualization of the geometry of the treatment network reflected by the network structure.	8
Study characteristic s	18	For each study, present characteristics for which data were extracted (e.g., study size, PICOS, follow-up period) and provide the citations.	8
Risk of bias within studies	19	Present data on risk of bias of each study and, if available, any outcome level assessment (see item 12).	figure S1
Results of individual studies	20	For all outcomes considered (benefits or harms), present, for each study:1) simple summary data for each intervention group, and 2) effect estimates and confidence/credible intervals. Modified approaches may be needed to deal with information from larger networks.	8
Synthesis of results	21	Present results of each meta-analysis done, including confidence/credible intervals. In larger networks, authors may focus on comparisons versus a particular comparator (e.g. placebo or standard care), with full findings presented in an appendix. League tables and forest plots may be considered to summarize pairwise comparisons. If additional summary measures were explored (such as treatment rankings), these should also be presented.	8-13
Exploration for inconsistency	S5	Describe results from investigations of inconsistency. This may include such information as measures of model fit to compare consistency and inconsistency models, P values from statistical tests, or summary of inconsistency estimates from different parts of the treatment network.	12-13
Risk of bias across studies	22	Present results of any assessment of risk of bias across studies (see Item 15).	13
Additional analysis	23	Give results of additional analyses, if done (e.g., sensitivity or subgroup analyses, meta-regression, alternative network geometries studied, alternative choice of prior distributions for Bayesian analyses, and so forth [see Item 16])	13
**DISCUSSION**			
Summary of evidence	24	Summarize the main findings including the strength of evidence for each main outcome; consider their relevance to key groups (e.g., healthcare providers, users, and policy makers).	13-17
Limitations	25	Discuss limitations at study and outcome level (e.g., risk of bias), and at review-level (e.g., incomplete retrieval of identified research, reporting bias). Comment on the validity of the assumptions, such as transitivity and consistency. Comment on any concerns regarding network geometry (e.g., avoidance of certain comparisons).	17-18
Conclusions	26	Provide a general interpretation of the results in the context of other evidence, and implications for future research.	18

FUNDING			
Funding	27	Describe sources of funding for the systematic review and other support (e.g., supply of data); role of funders for the systematic review.	18

PRISMA = Preferred Reporting Items for Systematic Reviews and Meta-Analysis; PICOS = population, intervention, comparators, outcomes, study design.

*Text in italics indicates wording specific to reporting of network meta-analyses that has been added to guidance from the PRISMA statement.

Table S2. Literature search criteria

#1--406,272
gastric[Title/Abstract] OR stomach[Title/Abstract] OR 'gastro-esophageal junction'[Title/Abstract] OR oesophagogastric[Title/Abstract] OR oesophagastric[Title/Abstract] OR esophagogastric[Title/Abstract] OR esophago-gastric[Title/Abstract] OR gastroesophageal[Title/Abstract] OR gastro-oesophageal[Title/Abstract]
#2--60,370
Programmed Death-1[Title/Abstract] OR PD 1 Inhibitor[Title/Abstract] OR CTLA 4 Inhibitor[Title/Abstract] OR Pd-1[Title/Abstract] OR pd1[Title/Abstract] OR Programmed Death Ligand-1[Title/Abstract] OR PD L1 Inhibitor[Title/Abstract] OR Pd-l1[Title/Abstract] OR Pdl1[Title/Abstract] OR Checkpoint Inhibitor[Title/Abstract] OR Immune Checkpoint Inhibitor[Title/Abstract] OR Checkpoint Blockade[Title/Abstract]
#3--2,709,381
carcinoma[Title/Abstract] OR cancer[Title/Abstract] OR adenocarcinoma[Title/Abstract]
#4--10,617,347
randomized controlled trial[Title/Abstract] OR controlled clinical trial[Title/Abstract] OR clinical trials as topic[Title/Abstract] OR random allocation[Title/Abstract] OR double-blind method[Title/Abstract] OR single-blind method[Title/Abstract] OR open label[Title/Abstract] OR clinical trial[Title/Abstract] OR clinical[Title/Abstract] OR research[Title/Abstract] OR comparative study[Title/Abstract] OR evaluation studies[Title/Abstract] OR follow-up[Title/Abstract] OR prospective[Title/Abstract] OR mask[Title/Abstract] OR blind[Title/Abstract] OR placebo[Title/Abstract] OR random[Title/Abstract] OR control[Title/Abstract] OR controls[Title/Abstract] OR prospectiv[Title/Abstract] OR volunteer[Title/Abstract]
#5--1,163
#1 AND #2 AND #3 AND #4 AND 2013/01/01[PDAT]:2024/05/08[PDAT]

Table S3. Bayesian ranking results of network meta-analysis.

Treatment	Rank of possibility (%)	

	1	2	3	4	5	6	7	8
**Overall survival for ITT population**								
**Chemo**	0	0	0	0	1	13	86	-
**Tisl-chemo**	1	18	25	24	23	8	1	-
**Nivo-chemo**	1	8	19	31	34	7	0	-
**Nivo-ipi**	0	1	3	7	14	62	13	-
**Pemb-chemo**	2	27	33	23	12	3	0	-
**Sint-chemo**	7	38	17	15	16	7	0	-
**Cado-chemo**	89	8	3	0	0	0	0	-
**Progression free survival for ITT population**								
**Chemo**	0	0	0	0	1	99	0	-
**Tisl-chemo**	0	4	26	26	43	1	0	-
**Nivo-chemo**	0	2	28	38	32	0	0	-
**Nivo-ipi**	0	0	0	0	0	0	100	-
**Pemb-chemo**	0	6	39	32	23	0	0	-
**Sint-chemo**	9	79	7	4	1	0	0	-
**Cado-chemo**	91	9	0	0	0	0	0	-
**Overall survival for participants receiving chemotherapy with XELOX**								
**Chemo**	0	0	0	0	1	99	-	-
**Tisl-chemo**	2	18	26	29	25	0	-	-
**Nivo-chemo**	1	9	17	29	44	0	-	-
**Pemb-chemo**	3	34	33	20	10	0	-	-
**Sint-chemo**	6	31	21	21	20	1	-	-
**Cado-chemo**	88	8	3	1	0	0	-	-
**Overall survival for Asian patients**								
**Chemo**	0	0	0	0	3	97	-	-
**Tisl-chemo**	1	6	18	32	42	1	-	-
**Nivo-chemo**	1	7	23	35	33	1	-	-
**Pemb-chemo**	15	48	21	10	5	1	-	-
**Sint-chemo**	5	22	34	22	17	0	-	-
**Cado-chemo**	78	17	4	1	0	0	-	-
**Overall survival for CPS ≥1**								
**Chemo**	0	0	0	1	4	95	-	-
**Nivo-chemo**	3	36	39	19	4	0	-	-
**Nivo-ipi**	1	2	6	16	71	4	-	-
**Pemb-chemo**	1	12	32	45	11	0	-	-
**Sint-chemo**	9	41	21	19	9	0	-	-
**Cado-chemo**	88	9	2	1	0	0	-	-
**Overall survival for CPS ≥5**								
**Chemo**	0	0	0	1	15	84	-	-
**Nivo-chemo**	4	28	45	20	2	0	-	-
**Nivo-ipi**	0	1	3	14	67	15	-	-
**Sint-chemo**	17	44	22	14	3	0	-	-

**Cado-chemo**	77	14	5	3	1	0	-	-
**Suge-chemo**	2	13	25	48	12	0	-	-
**Overall survival for CPS ≥10**								
**Chemo**	0	0	0	0	0	9	91	-
**Nivo-chemo**	2	12	30	33	21	2	0	-
**Nivo-ipi**	0	0	1	2	10	78	9	-
**Pemb-chemo**	1	8	21	32	35	3	0	-
**Sint-chemo**	31	40	14	8	6	1	0	-
**Cado-chemo**	58	21	8	7	5	1	0	-
**Suge-chemo**	7	19	26	19	23	5	0	-
**Grade ≥3 treatment-related adverse events**								
**Chemo**	0	0	1	3	14	36	44	3
**Tisl-chemo**	6	8	12	15	19	2	15	7
**Nivo-chemo**	36	33	17	8	4	2	1	0
**Nivo-ipi**	1	1	1	2	3	5	11	77
**Pemb-chemo**	5	12	23	26	2	8	4	1
**Sint-chemo**	11	15	22	19	15	9	7	3
**Cado-chemo**	38	25	14	1	6	4	2	1
**Suge-chemo**	5	27	11	17	19	17	17	7
**Adverse events leading to discontinuation**								
**Chemo**	0	0	0	2	8	32	48	0
**Tisl-chemo**	8	37	22	15	9	5	3	2
**Nivo-chemo**	2	15	29	27	16	7	3	2
**Nivo-ipi**	1	1	2	3	5	9	18	61
**Pemb-chemo**	2	7	14	26	33	13	4	2
**Sint-chemo**	6	23	21	16	15	9	6	4
**Cado-chemo**	81	11	4	2	1	1	1	0
**Suge-chemo**	2	5	8	1	13	24	18	19

TT: intent-to-treat; Pemb, pembrolizumab; Tisl-chemo, tislelizumab plus chemotherapy; Nivo-chemo, nivolumab plus chemotherapy; Nivo-ipi, nivolumab plus ipilimumab; Pemb-chemo, pembrolizumab plus chemotherapy; Sint-chemo, sintilimab plus chemotherapy; Cado-chemo, cadonilimab plus chemotherapy; Suge-chemo, sugemalimab plus chemotherapy; Chemo, chemotherapy

Table S4. Probability of occurrence of a specific adverse event.

	**Platelet count decreased**	**Neutrophil count decreased**	**WBC count decreased**	**Anemia**
**Tisl-chemo**	34%	32%	21%	33%
**Nivo-chemo**	26%	28%	17%	24%
**Nivo-ipi**	1%	1%	1%	7%
**Pemb-chemo**	21%	24%	13%	32%
**Sint-chemo**	66%	59%	54%	47%

**Cado-chemo**	66%	60%	51%	49%
**Suge-chemo**	53%	52%	50%	62%
	**AST increased**	**ALT increased**	**Gamma-glutamyl transferase increased**	**Blood bilirubin increased**
**Tisl-chemo**	28%	21%	<20%	<20%
**Nivo-chemo**	16%	11%	<10%	<10%
**Nivo-ipi**	13%	13%	<10%	<10%
**Pemb-chemo**	18%	13%	<10%	10%
**Sint-chemo**	33%	22%	<15%	17%
**Cado-chemo**	35%	22%	<20%	<20%
**Suge-chemo**	0.31	22%	<20%	<20%
	**Blood creatinine increased**	**Hypothyroidis m**	**Palmar-plantar erythrodysaesthesi a**	**Peripheral sensory neuropathy**
**Tisl-chemo**	<20%	<20%	<20%	23%
**Nivo-chemo**	<10%	<10%	13%	30%
**Nivo-ipi**	<10%	12%	21%	0%
**Pemb-chemo**	<10%	13%	24%	17%
**Sint-chemo**	<15%	18%	17%	15%
**Cado-chemo**	<20%	<20%	<20%	<20%
**Suge-chemo**	<20%	<20%	<20%	<20%

Tisl-chemo, tislelizumab plus chemotherapy; Nivo-chemo, nivolumab plus chemotherapy; Pemb-chemo, pembrolizumab plus chemotherapy; Sint-chemo, sintilimab plus chemotherapy; cado-chemo, cadonilimab plus chemotherapy; Suge-chemo, sugemalimab plus chemotherapy

Table S5. Comparisons of the fit of consistency and inconsistency models using deviance information criteria (DIC)

	ITT population				
Model	Overall survival	Progression-free survival	Grade ≥3 adverse events	AEs leading to discontinuation	
Consistency	-9.2	-10.1	0.17	0.24	
Inconsistency	-8.7	-8.8	0.31	0.74	
	XELOX	Asian	PD-L1 CPS ≥1	PD-L1 CPS ≥5	PD-L1 CPS ≥10
Model	Overall survival	Overall survival	Overall survival	Overall survival	Overall survival
Consistency	-7.4	-1.7	-6.5	-2.3	-2.6
Inconsistency	-5.9	-3.7	-6.1	-2.3	-1.5

The DIC is a Bayesian model evaluation criterion that measures model fit adjusted with complexity of the model; smaller DIC values correspond to more preferable models. (Reference: Spiegelhalter, D.J., Best, N.G., Carlin, B.P., Van der Linde, A. Bayesian measures of model complexity and fit. Journal of the Royal Statistical Society Series B (Statistical Methodology) 2002; 64(4):583-639).

Table S6. Bayesian ranking results of the sensitivity analysis (excluding the treatment of nivo-ipi).

**Treatment**	**Rank of possibility (%)**						
	**1**	**2**	**3**	**4**	**5**	**6**	**7**
**Overall survival for ITT population (sensitivity analysis)**							
**Chemo**	0	0	0	0	1	99	-
**Tisl-chemo**	1	19	25	26	29	0	-
**Nivo-chemo**	0	7	20	35	37	1	-
**Pemb-chemo**	2	29	34	23	12	0	-
**Sint-chemo**	7	38	19	15	21	0	-
**Cado-chemo**	90	7	2	1	0	0	-
**Progression free survival for ITT population (sensitivity analysis)**							
**Chemo**	0	0	0	0	1	99	-
**Tisl-chemo**	0	4	26	26	43	1	-
**Nivo-chemo**	0	3	27	39	31	0	-
**Pemb-chemo**	0	5	39	32	24	0	-
**Sint-chemo**	9	80	7	3	1	0	-
**Cado-chemo**	91	8	1	0	0	0	-
**Grade >=3 treatment-related adverse events (sensitivity analysis)**							
**Chemo**	0	0	1	4	15	37	44
**Tisl-chemo**	5	7	11	15	20	23	18
**Nivo-chemo**	38	31	17	8	4	1	1
**Pemb-chemo**	5	12	22	32	17	8	3
**Sint-chemo**	10	14	24	19	15	10	7
**Cado-chemo**	37	29	14	9	5	4	2
**Suge-chemo**	5	7	10	13	24	18	24
**Adverse events leading to discontinuation (sensitivity analysis)**							
**Chemo**	0	0	1	3	10	39	48
**Tisl-chemo**	8	37	24	14	9	5	3
**Nivo-chemo**	2	14	29	29	16	7	3
**Pemb-chemo**	1	7	15	27	32	14	4
**Sint-chemo**	6	27	19	16	17	9	7
**Cado-chemo**	82	10	4	2	1	1	1
**Suge-chemo**	2	5	7	10	15	26	34
**Overall survival for PL-L1 CPS ≥1 (sensitivity analysis)**							
**Chemo**	0	0	0	1	99	-	-

**Nivo-chemo**	3	36	41	20	1	-	-
**Pemb-chemo**	1	13	35	52	0	-	-
**Sint-chemo**	9	42	22	27	0	-	-
**Cado-chemo**	88	9	2	1	0	-	-
**Overall survival for PL-L1 CPS ≥5 (sensitivity analysis)**							
**Chemo**	0	0	0	1	99	-	-
**Nivo-chemo**	4	29	46	21	0	-	-
**Sint-chemo**	17	45	22	17	1	-	-
**Cado-chemo**	77	14	5	3	1	-	-
**Suge-chemo**	1	13	27	59	0	-	-
**Overall survival for PL-L1 CPS ≥10 (sensitivity analysis)**							
**Chemo**	0	0	0	0	1	99	-
**Nivo-chemo**	2	13	30	33	22	0	-
**Pemb-chemo**	0	8	22	33	36	0	-
**Sint-chemo**	32	39	15	8	6	0	-
**Cado-chemo**	58	21	8	5	7	0	-
**Suge-chemo**	7	20	26	20	27	0	-

ITT: intent-to-treat; Tisl-chemo, tislelizumab plus chemotherapy; Nivo-chemo, nivolumab plus chemotherapy; Pemb-chemo, pembrolizumab plus chemotherapy; Sint-chemo, sintilimab plus chemotherapy; Cado-chemo, cadonilimab plus chemotherapy; Suge-chemo, sugemalimab plus chemotherapy; Chemo, chemotherapy

A. History of overall survival for ITT population

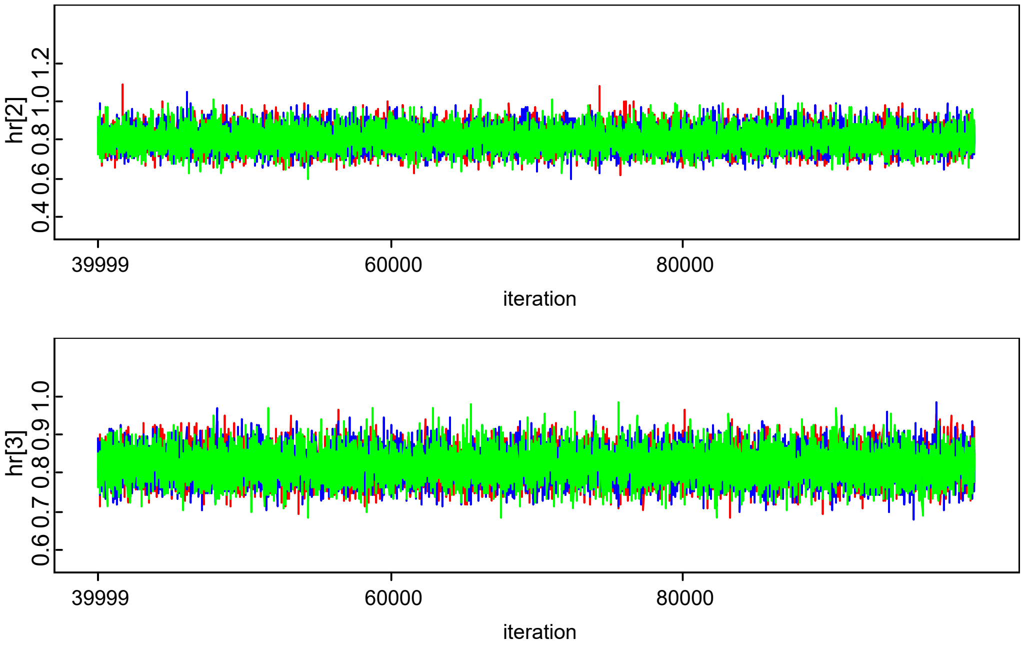

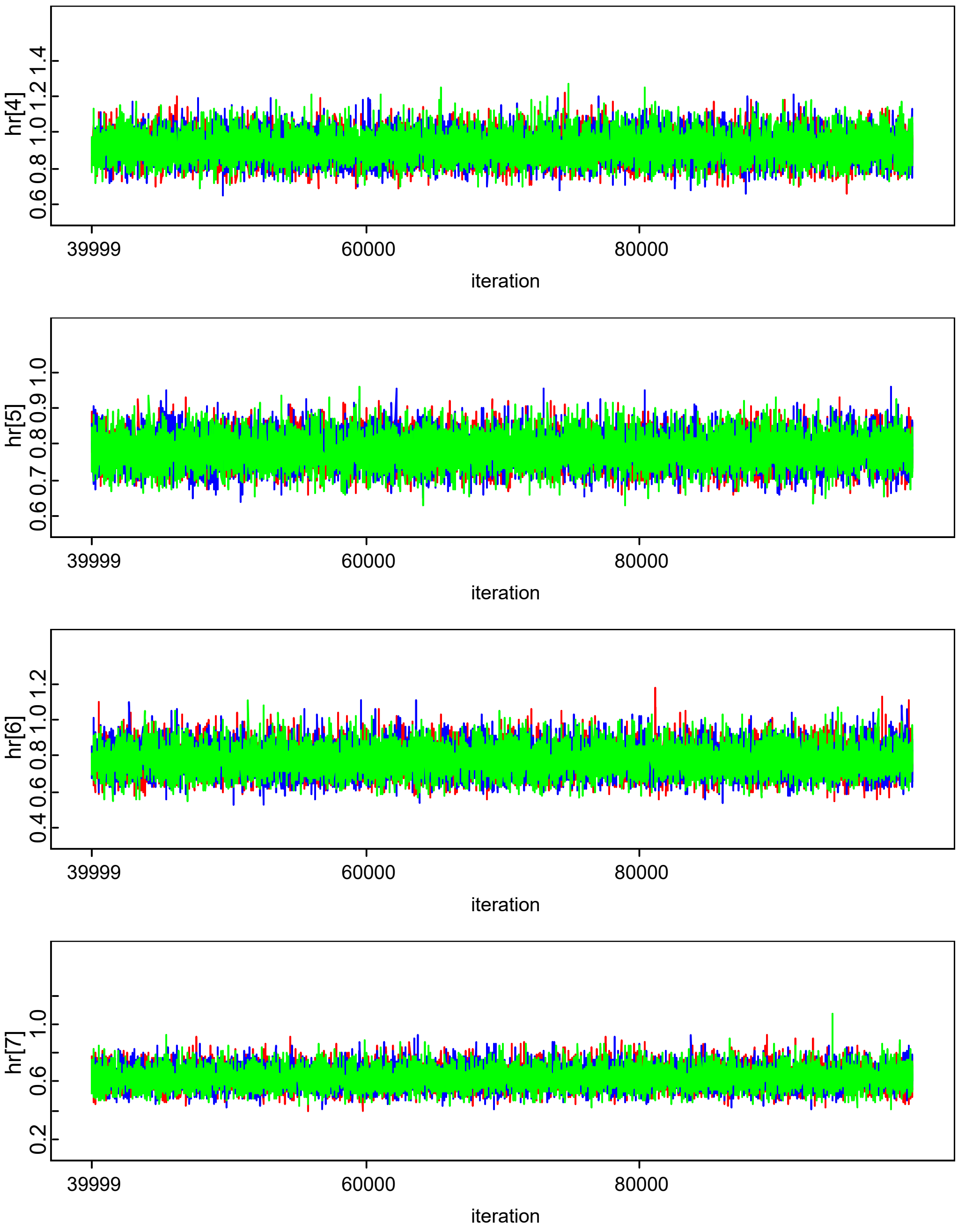

B. Density of overall survival for ITT population

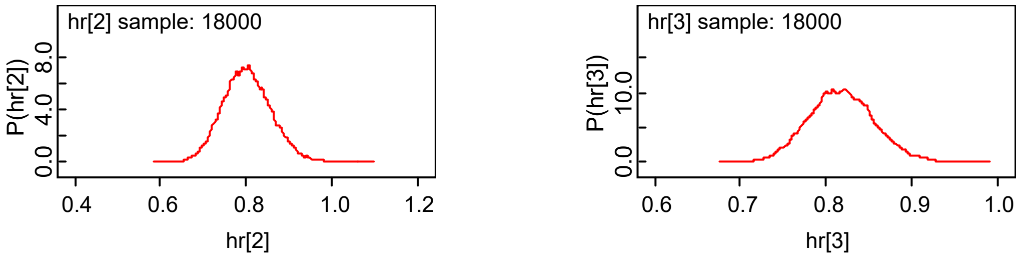

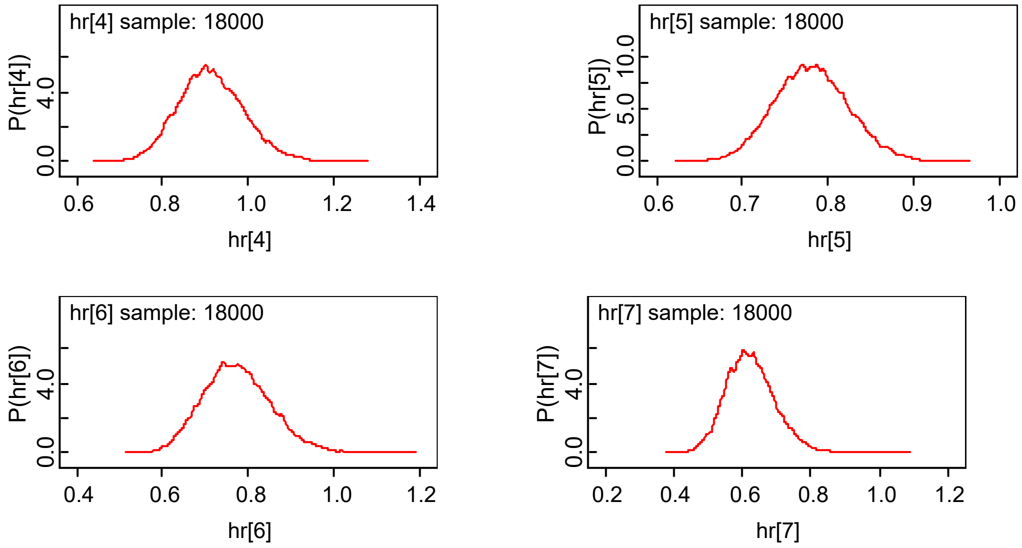

C. History of progression free survival for ITT population

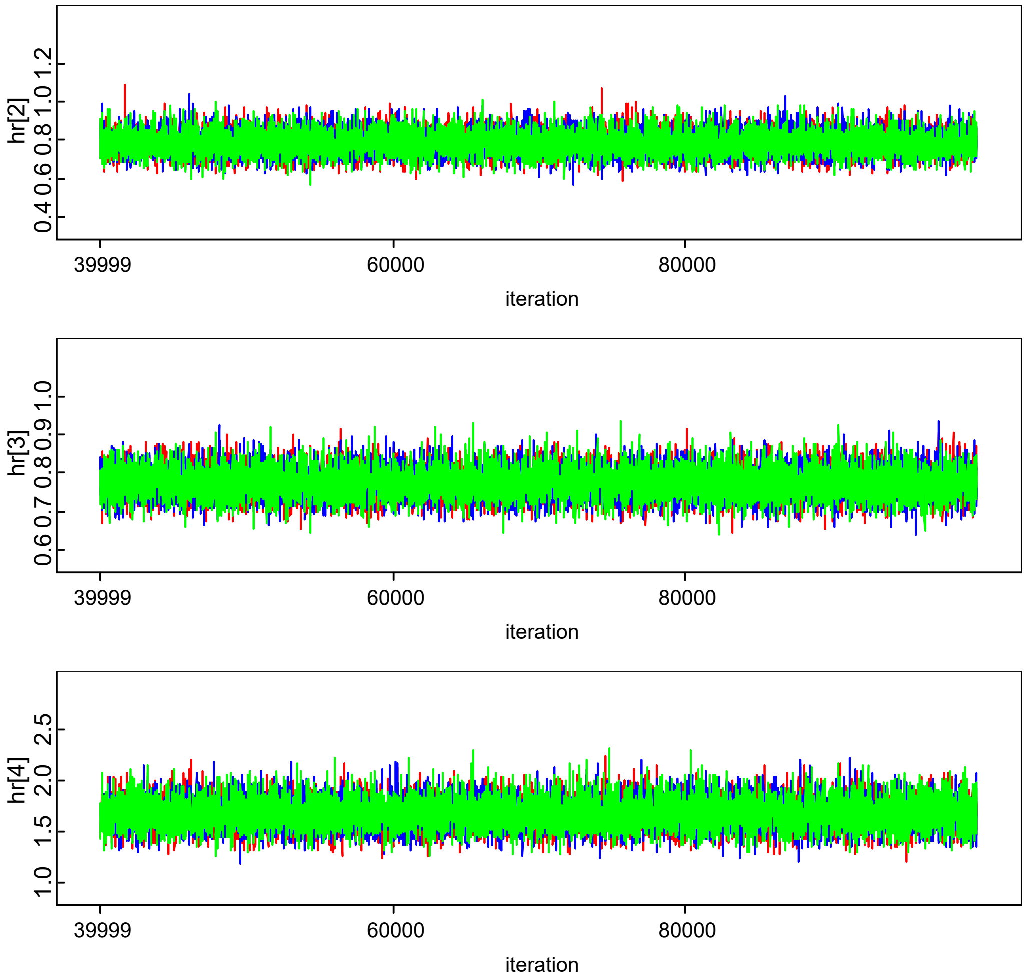

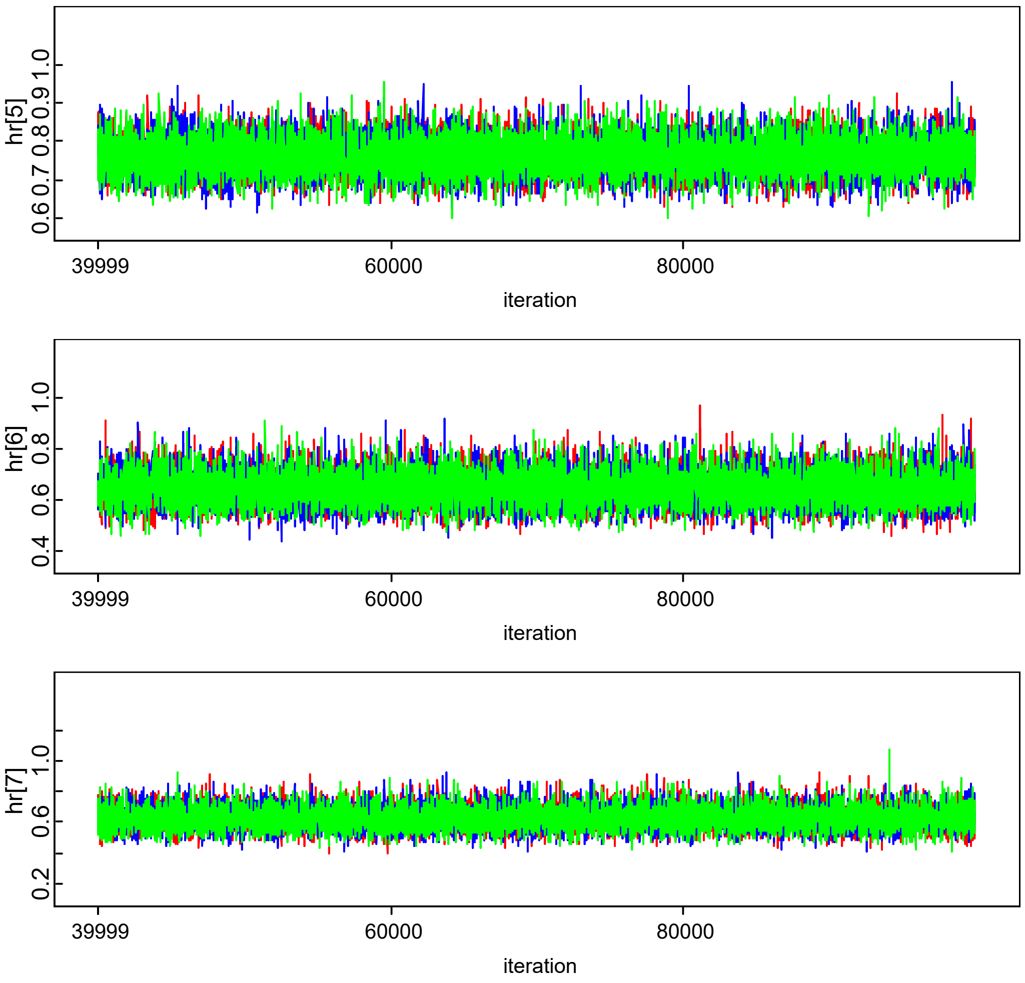

D. Density of progression free survival for ITT population

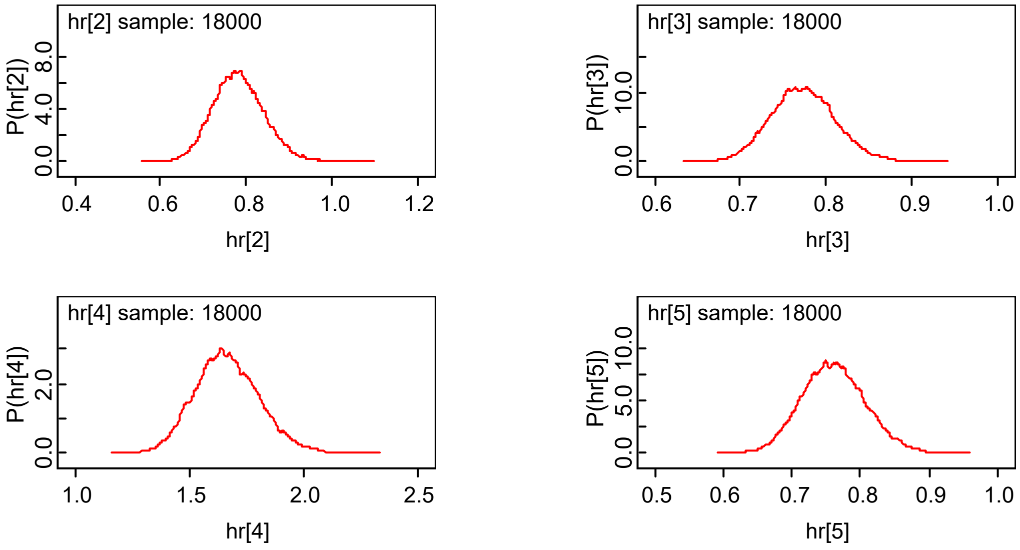

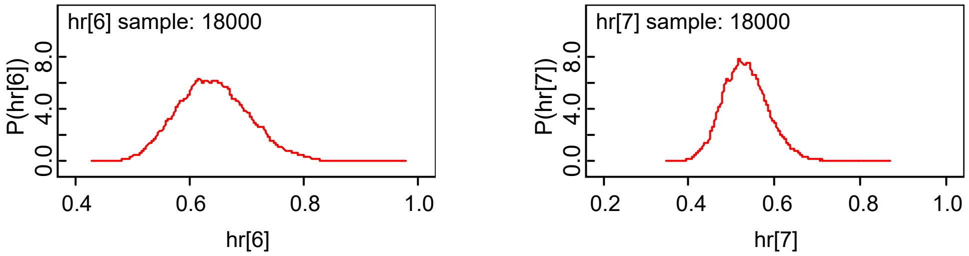

Figure S1. Convergence of the three Markov Chain Monte Carlo chains established by of the history feature for overall survival and progression-free survival. 2: tisl-chemo; 3: nivo-chemo; 4: nivo-ipi; 5: pemb-chemo; 6: sint-chemo; 7: cado-chemo

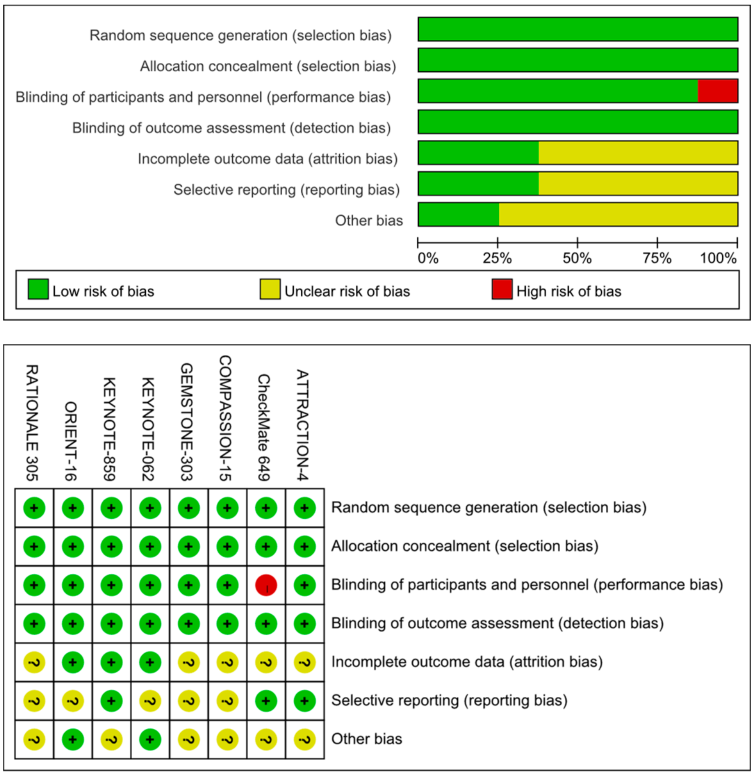

Figure S2. Summary of results from assessment of studies using the Cochrane risk of bias tool.

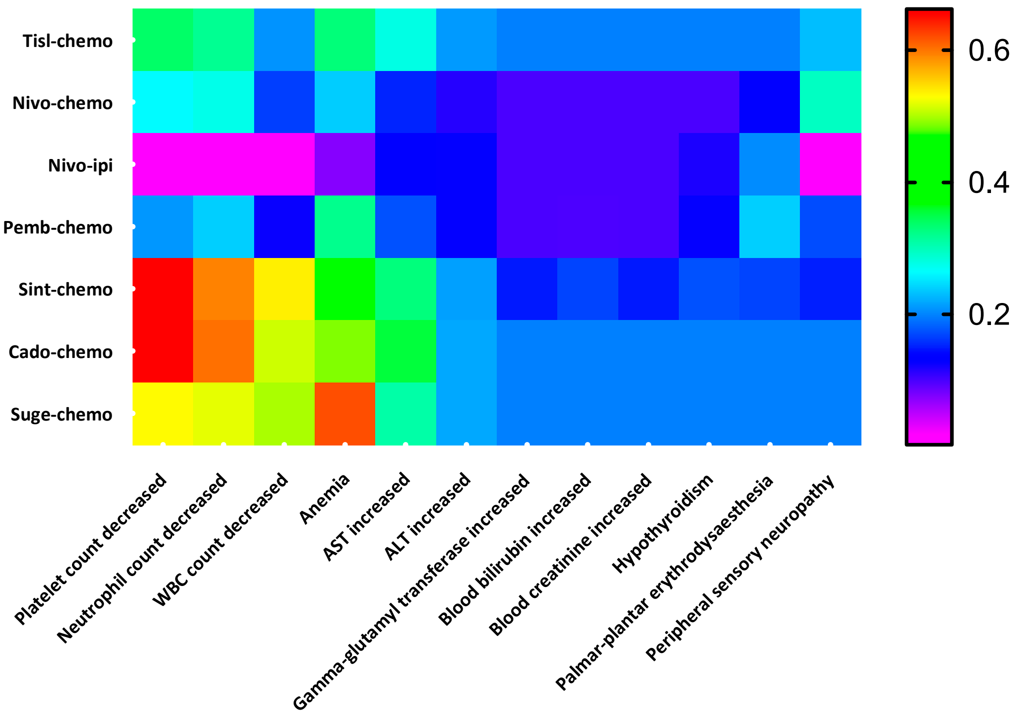

Figure S3. The probability of a specific adverse event is shown as a heat map. The pure decimal on the right of the picture represents the probability of occurrence, with the decimal numbers increasing from bottom to top representing the probability of occurrence of a specific adverse event. Tisl-chemo, tislelizumab plus chemotherapy; Nivo-chemo, nivolumab plus chemotherapy; Nivo-ipi, nivolumab plus ipilimumab; Pemb-chemo, pembrolizumab plus chemotherapy; Sint-chemo, sintilimab plus chemotherapy; Cado-chemo, cadonilimab plus chemotherapy; Suge-chemo, sugemalimab plus chemotherapy

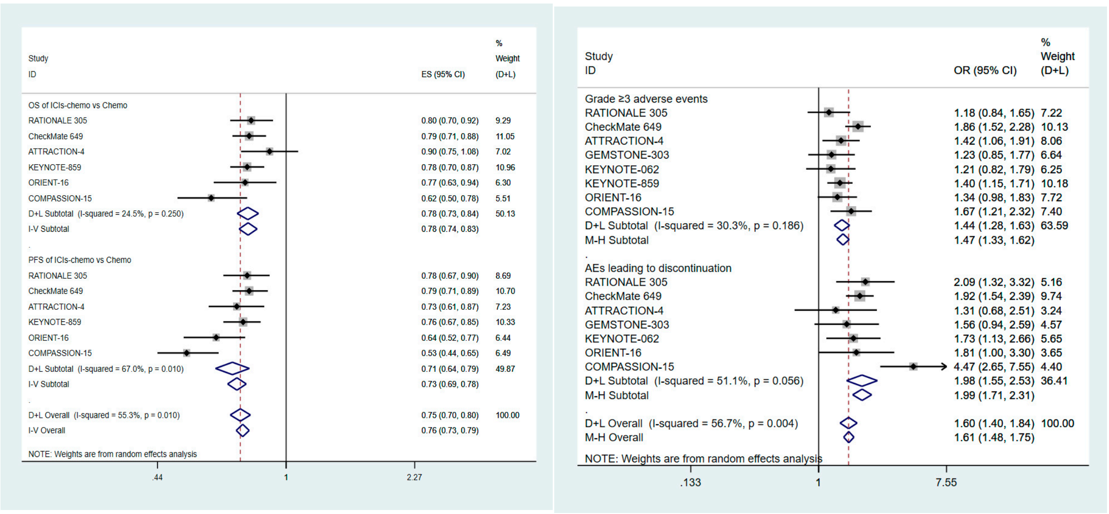

Figure S4. Forest plots depicting results of head-to-head comparisons according to pairwise meta-analyses on different outcomes.

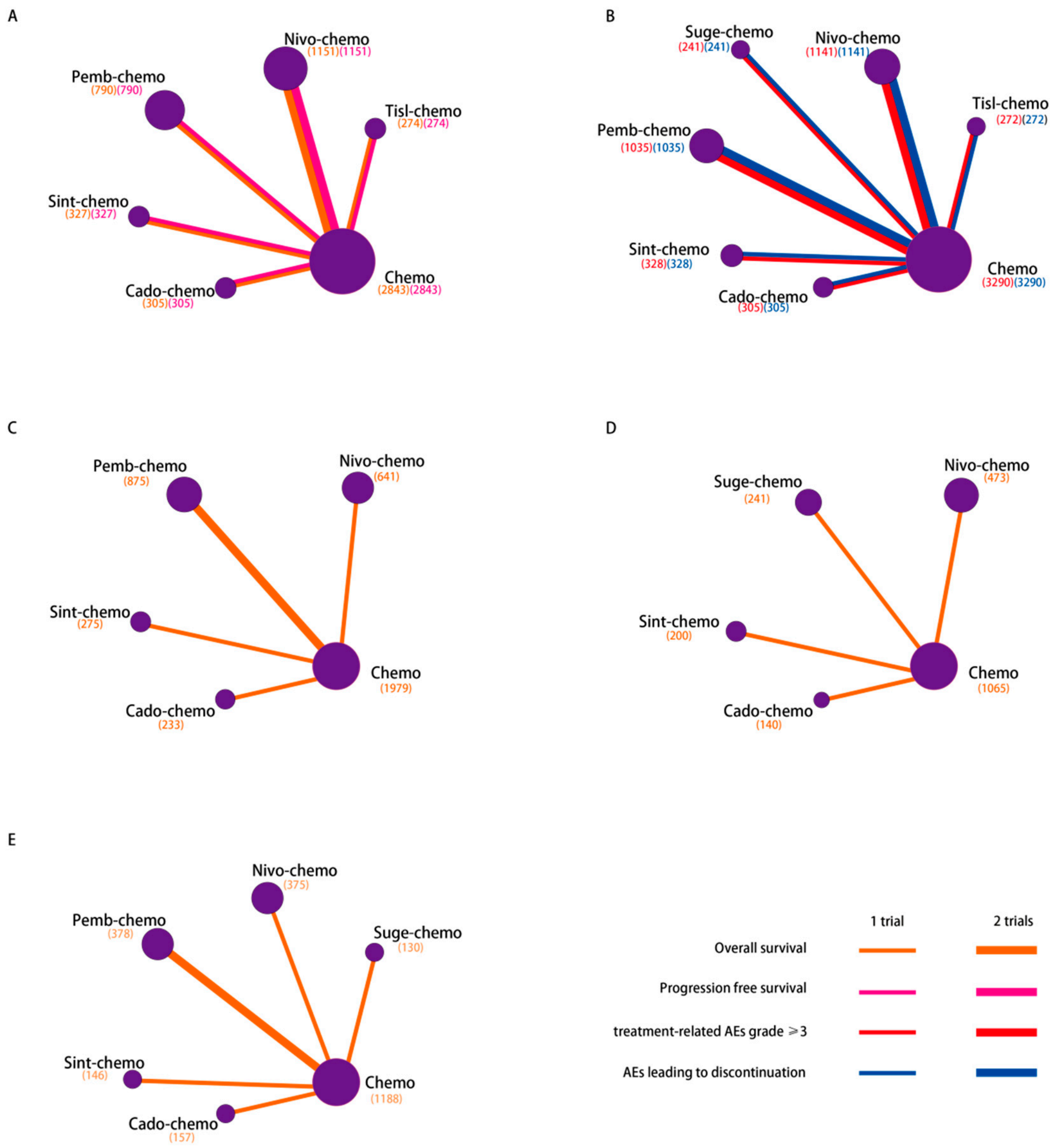

Figure S5. Network diagrams for the fist sensitivity analysis (exclude the treatment of nivo-ipi).

(A) Comparisons on overall survival and progression free survival in ITT population. (B) Comparisons on TRAEs of grade 3 or higher and AEs leading to discontinuation. (C) Comparison on overall survival in PD-L1 CPS ≥1. (D) Comparison on overall survival in PD-L1 CPS ≥5. (E) Comparison on overall survival in PD-L1 CPS ≥10. TRAE, treatment related adverse events; Tisl-chemo, tislelizumab plus chemotherapy; Nivo-chemo, nivolumab plus chemotherapy; Pemb-chemo, pembrolizumab plus chemotherapy; Sint-chemo, sintilimab plus chemotherapy; Cado-chemo, cadonilimab plus chemotherapy; Suge-chemo, sugemalimab plus chemotherapy; Chemo, chemotherapy

A Overall survival (the fist sensitivity analysis)

Chemo	**0.80 (0.70 to 0.92)**	**0.82 (0.75 to 0.90)**	**0.78 (0.70 to 0.87)**	**0.77 (0.63 0.94)**	**0.62 (0.50 to 0.77)**
**1.28 (1.11 to 1.49)**	Tisl-chemo	1.02 (0.87 to 1.21)	0.98 (0.82 to 1.17)	0.96 (0.75 to 1.23)	0.77 (0.60 to 1.01)
**1.30 (1.18 to 1.43)**	1.01 (0.85 to 1.21)	Nivo-chemo	0.95 (0.83 to 1.10)	0.94 (0.76 to 1.18)	**0.76 (0.60 to 0.96)**
**1.32 (1.17 to 1.46)**	1.03 (0.85 to 1.24)	1.02 (0.87 to 1.18)	Pemb-chemo	0.99 (0.79 to 1.24)	0.79 (0.62 to 1.02)
**1.56 (1.29 to 1.90)**	1.22 (0.95 to 1.56)	1.21 (0.97 to 1.50)	1.19 (0.94 to 1.49)	Sint-chemo	0.80 (0.60 to 1.08)
**1.88 (1.55 to 2.30)**	**1.47 (1.15 to 1.89)**	**1.46 (1.17 to 1.82)**	**1.43 (1.14 to 1.81)**	**1.21 (0.92 to 1.60)**	Cado-chemo

Progression free survival (the fist sensitivity analysis)

B Grade >=3 treatment-related adverse events (the fist sensitivity analysis)

Chemo	1.15 (0.62, 2.23)	**1.69 (1.06, 2.50)**	1.34 (0.85, 2.07)	1.38 (0.73, 2.52)	1.71 (0.89, 3.15)	1.15 (0.61, 2.18)
0.48 (0.15, 1.49)	Tisl-chemo	1.46 (0.64, 2.94)	1.16 (0.52, 2.46)	1.18 (0.46, 2.79)	1.47 (0.58, 3.44)	0.99 (0.40, 2.40)
0.56 (0.28, 1.40)	1.21 (0.32, 5.47)	Nivo-chemo	0.79 (0.44, 1.50)	0.79 (0.39, 1.77)	0.98 (0.47, 2.20)	0.66 (0.33, 1.53)
0.65 (0.29, 1.42)	1.39 (0.35, 5.49)	1.16 (0.31, 3.12)	Pemb-chemo	1.03 (0.47, 2.17)	1.27 (0.59, 2.74)	0.85 (0.40, 1.86)
0.54 (0.16, 1.77)	1.13 (0.22, 5.90)	0.94 (0.21, 3.66)	0.82 (0.19, 3.51)	Sint-chemo	1.26 (0.50, 2.97)	0.82 (0.35, 2.08)
**0.23 (0.07, 0.71)**	0.47 (0.09, 2.47)	0.39 (0.09, 1.47)	0.34 (0.09, 1.45)	0.41 (0.08, 2.23)	Cado-chemo	0.67 (0.28, 1.72)
0.88 (0.26, 2.94)	1.84 (0.36, 9.74)	1.56 (0.32, 5.88)	1.34 (0.32, 5.81)	1.58 (0.30, 9.03)	3.86 (0.76, 21.38)	Suge-chemo

Adverse events leading to discontinuation (the fist sensitivity analysis)

C Overall survival (PD-L1 CPS ≥1, the fist sensitivity analysis)

Chemo	**0.74 (0.65 to 0.83)**	**0.77 (0.70 to 0.86)**	**0.73 (0.58 to 0.91)**	**0.58 (0.44 to 0.75)**	
**1.43 (1.24 to 1.65)**	Nivo-chemo	1.05 (0.89 to 1.23)	0.99 (0.77 to 1.26)	0.78 (0.58 to 1.05)	
		Pemb-chemo	0.94 (0.74 to 1.20)	**0.75 (0.56 to 0.99)**	
**1.51 (1.16 to 1.99)**	1.06 (0.78 to 1.44)		Sint-chemo	0.79 (0.56 to 1.12)	
**1.85 (1.30 to 2.63)**	1.30 (0.89 to 1.89)		1.22 (0.79 to 1.89)	Cado-chemo	
**1.33 (1.09 to 1.63)**	0.93 (0.73 to 1.20)		0.88 (0.63 to 1.24)	0.72 (0.48 to 1.07)	Suge-chemo

Overall survival (PD-L1 CPS ≥5, the fist sensitivity analysis)

D **Overall survival (PD-L1 CPS ≥10, the fist sensitivity analysis)**

Chemo	**0.66 (0.56 to 0.78)**	**0.68 (0.58 to 0.80)**	**0.56 (0.41 to 0.77)**	**0.51 (0.33 to 0.80)**	**0.65 (0.49 to 0.85)**
	Nivo-chemo	1.03 (0.82 to 1.31)	0.85 (0.60 to 1.21)	0.78 (0.48 to 1.25)	0.98 (0.72 to 1.35)
		Pemb-chemo	0.82 (0.58 to 1.18)	0.75 (0.46 to 1.22)	0.96 (0.69 to 1.32)
			Sint-chemo	0.91 (0.53 to 1.58)	1.16 (0.76 to 1.77)
				Cado-chemo	1.27 (0.75 to 2.18)
					Suge-chemo

Figure S6. Pooled estimates of the fist sensitivity analysis. (A) Upper triangular section: Hazard ratios (95% CrI) for overall survival (OS); Lower triangular section: Hazard ratios (95% CrI) for progression-free survival (PFS). (B) Upper triangular section: Odds ratios (95% CrI) for grade ≥3 adverse events; Lower triangular section: adverse events leading to discontinuation. (C) Upper triangular section: hazard ratios (95% CrI) for overall survival for patients with PD-L1 CPS ≥1; Lower triangular section: hazard ratios (95% CrI) for overall survival for patients with PD-L1 CPS ≥5. (D) Upper triangular section: hazard ratios (95% CrI) for overall survival for patients with PD-L1 CPS ≥10. The data within each cell represent the hazard ratios or odds ratios (with 95% CrI) for the comparison between the treatment defined by the row and by the column. A hazard ratio of less than 1 and an odds ratio of greater than 1 indicate a favorable

effect for the row-defining treatment. Significant results are highlighted in bold. ITT: intent-to-treat; Tisl-chemo, tislelizumab plus chemotherapy; Nivo-chemo, nivolumab plus chemotherapy; Pemb-chemo, pembrolizumab plus chemotherapy; Sint-chemo, sintilimab plus chemotherapy; Cado-chemo, cadonilimab plus chemotherapy; Suge-chemo, sugemalimab plus chemotherapy; Chemo, chemotherapy

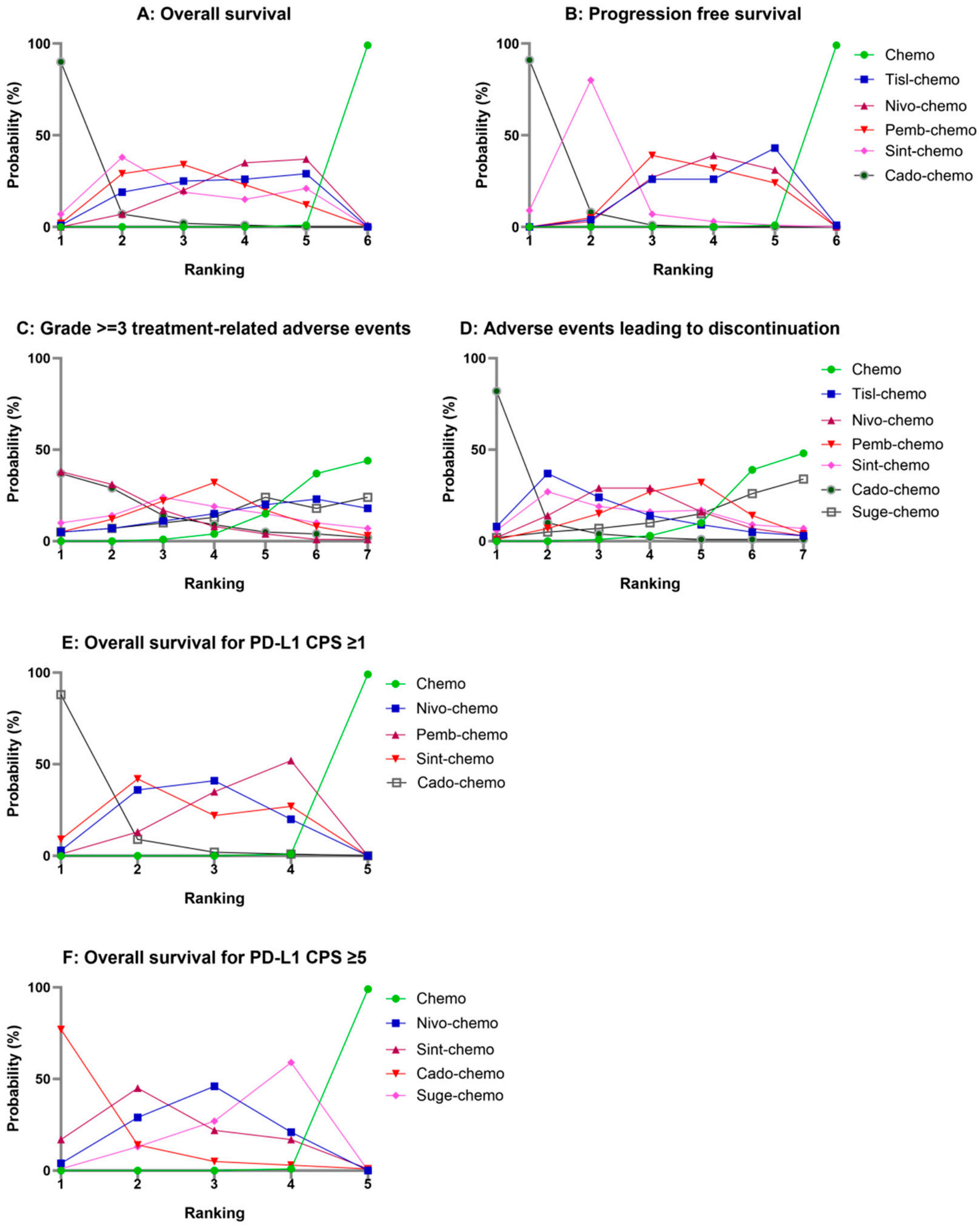

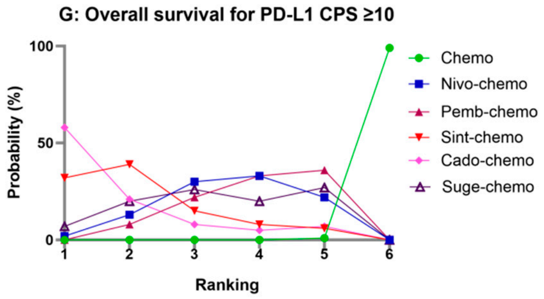

Figure S7. Bayesian ranking profiles of comparable treatments on efficacy and toxicity for the fist sensitivity analysis. Profiles indicate the probability of each comparable treatment being ranked from first to last on overall survival, progression free survival, grade ≥3 treatment-related adverse events, and adverse events leading to treatment discontinuation. Ranking curves are described according to the bayesian ranking results presented in supplementary table S6. Tisl-chemo, tislelizumab plus chemotherapy; Nivo-chemo, nivolumab plus chemotherapy; Pemb-chemo, pembrolizumab plus chemotherapy; Sint-chemo, sintilimab plus chemotherapy; Cado-chemo, cadonilimab plus chemotherapy; Suge-chemo, sugemalimab plus chemotherapy; Chemo, chemotherapy

A

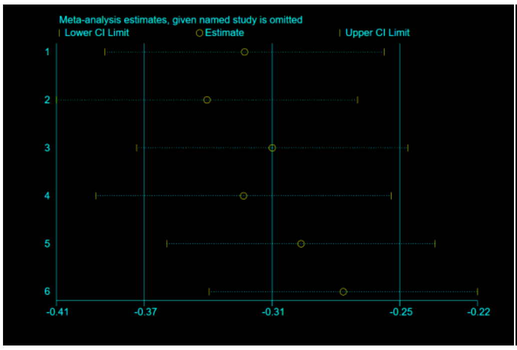

B

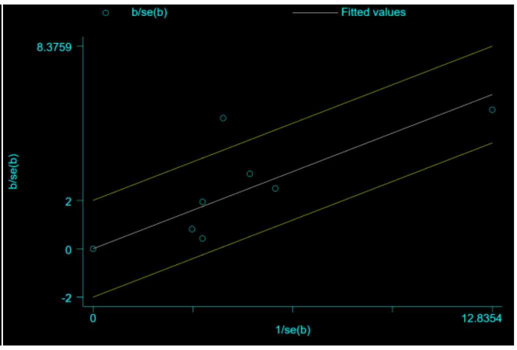

Figure S8. Leave-one-out sensitivity analyses was conducted to assess the impact of iteratively removing each individual trial. (A) Leave-one-out sensitivity analysis for progression-free survival; (B) Leave-one-out sensitivity analysis for adverse events leading to treatment discontinuation.

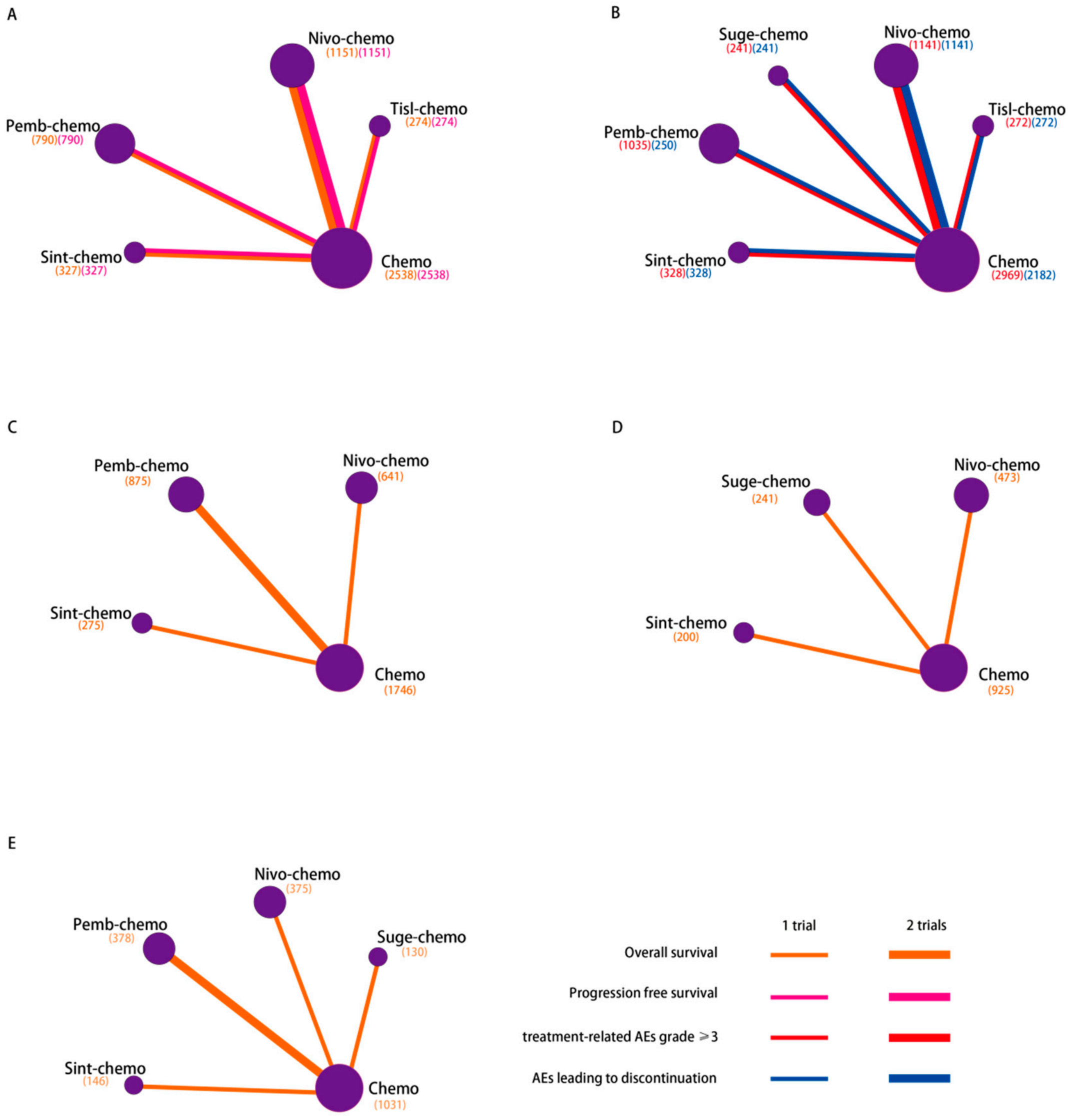

Figure S9. Network diagrams for the second sensitivity analysis (exclude the treatment of nivo-ipi and the trial of COMPASSION-15). (A) Comparisons on overall survival and progression free survival in ITT population. (B) Comparisons on TRAEs of grade 3 or higher and AEs leading to discontinuation. TRAE, treatment related adverse events.

A Overall survival (the second sensitivity analysis)

**Chemo**	**0.80 (0.70 to 0.92)**	**0.82 (0.75 to 0.90)**	**0.78 (0.70 to 0.87)**	**0.77 (0.63 to 0.94)**
**1.28 (1.11 to 1.49)**	**Tisl-chemo**	1.02 (0.87 to 1.21)	0.98 (0.82 to 1.17)	0.96 (0.75 to 1.23)

**1.30 (1.18 to 1.43)**	1.01 (0.85 to 1.21)	Nivo-chemo	0.95 (0.83 to 1.10)	0.94 (0.76 to 1.18)
**1.32 (1.17 to 1.46)**	1.03 (0.85 to 1.24)	1.02 (0.87 to 1.18)	Pemb-chemo	0.99 (0.79 to 1.24)
**1.56 (1.29 to 1.90)**	1.22 (0.95 to 1.56)	1.21 (0.97 to 1.50)	1.19 (0.94 to 1.49)	Sint-chemo

Progression free survival (the second sensitivity analysis)

B Grade >=3 treatment-related adverse events (the second sensitivity analysis))

Chemo	1.15 (0.62, 2.23)	**1.69 (1.06, 2.50)**	1.34 (0.85, 2.07)	1.38 (0.73, 2.52)	1.15 (0.61, 2.18)
0.48 (0.15, 1.49)	Tisl-chemo	1.46 (0.64, 2.94)	1.16 (0.52, 2.46)	1.18 (0.46, 2.79)	0.99 (0.40, 2.40)
0.56 (0.28, 1.40)	1.21 (0.32, 5.47)	Nivo-chemo	0.79 (0.44, 1.50)	0.79 (0.39, 1.77)	0.66 (0.33, 1.53)
0.65 (0.29, 1.42)	1.39 (0.35, 5.49)	1.16 (0.31, 3.12)	Pemb-chemo	1.03 (0.47, 2.17)	0.85 (0.40, 1.86)
0.54 (0.16, 1.77)	1.13 (0.22, 5.90)	0.94 (0.21, 3.66)	0.82 (0.19, 3.51)	Sint-chemo	0.82 (0.35, 2.08)
0.88 (0.26, 2.94)	1.84 (0.36, 9.74)	1.56 (0.32, 5.88)	1.34 (0.32, 5.81)	1.58 (0.30, 9.03)	Suge-chemo

Adverse events leading to discontinuation (the second sensitivity analysis)

C Overall survival (PD-L1 CPS ≥1, the fist sensitivity analysis)

Chemo	**0.74 (0.65 to 0.83)**	**0.77 (0.70 to 0.86)**	**0.73 (0.58 to 0.91)**	
**1.43 (1.24 to 1.65)**	Nivo-chemo	1.05 (0.89 to 1.23)	0.99 (0.77 to 1.26)	
		Pemb-chemo	0.94 (0.74 to 1.20)	
**1.51 (1.16 to 1.99)**	1.06 (0.78 to 1.44)		Sint-chemo	
**1.33 (1.09 to 1.63)**	0.93 (0.73 to 1.20)		0.88 (0.63 to 1.24)	Suge-chemo

Overall survival (PD-L1 CPS ≥5, the fist sensitivity analysis)

D Overall survival (PD-L1 CPS ≥10, the second sensitivity analysis)

Chemo	**0.66 (0.56 to 0.78)**	**0.68 (0.58 to 0.80)**	**0.56 (0.41 to 0.77)**	**0.65 (0.49 to 0.85)**
	Nivo-chemo	1.03 (0.82 to 1.31)	0.85 (0.60 to 1.21)	0.98 (0.72 to 1.35)

**Pemb-chemo**	0.82 (0.58 to 1.18)	0.96 (0.69 to 1.32)
	**Sint-chemo**	1.16 (0.76 to 1.77)
		**Suge-chemo**

Figure S10. Pooled estimates of the second sensitivity analysis. (A) Upper triangular section: Hazard ratios (95% CrI) for overall survival (OS); Lower triangular section: Hazard ratios (95% CrI) for progression-free survival (PFS). (B) Upper triangular section: Odds ratios (95% CrI) for grade ≥3 adverse events; Lower triangular section: adverse events leading to discontinuation. (C) Upper triangular section: hazard ratios (95% CrI) for overall survival for patients with PD-L1 CPS ≥1; Lower triangular section: hazard ratios (95% CrI) for overall survival for patients with PD-L1 CPS ≥5. (D) Upper triangular section: hazard ratios (95% CrI) for overall survival for patients with PD-L1 CPS ≥10. The data within each cell represent the hazard ratios or odds ratios (with 95% CrI) for the comparison between the treatment defined by the row and by the column. A hazard ratio of less than 1 and an odds ratio of greater than 1 indicate a favorable effect for the row-defining treatment. Significant results are highlighted in bold. ITT: intent-to-treat; Tisl-chemo, tislelizumab plus chemotherapy; Nivo-chemo, nivolumab plus chemotherapy; Pemb-chemo, pembrolizumab plus chemotherapy; Sint-chemo, sintilimab plus chemotherapy; Suge-chemo, sugemalimab plus chemotherapy; Chemo, chemotherapy

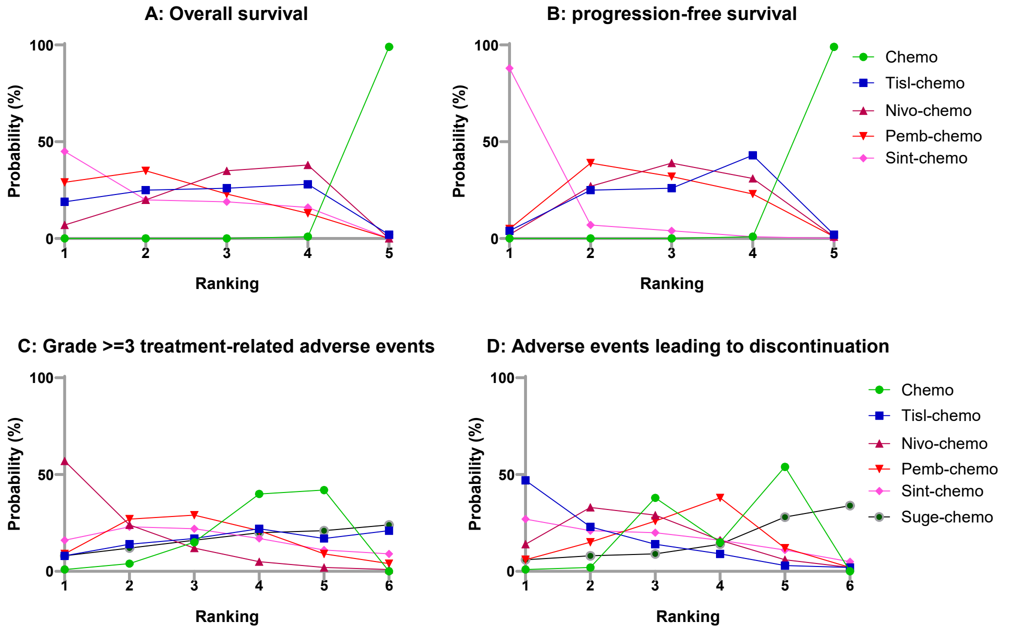

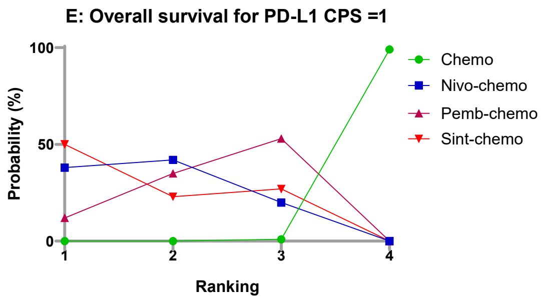

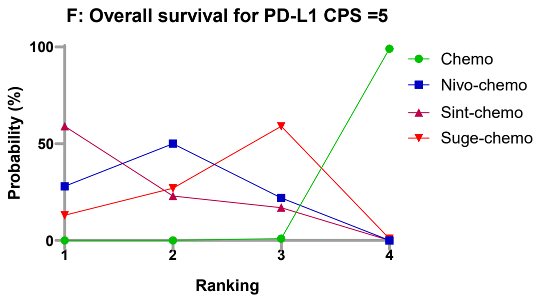

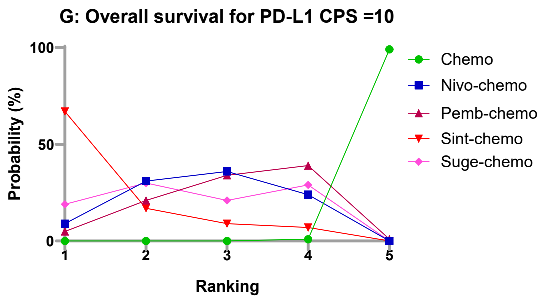

Figure S11. Bayesian ranking profiles of comparable treatments on efficacy and toxicity for the second sensitivity analysis. Profiles indicate the probability of each comparable treatment being ranked from first to last on overall survival, progression free survival, grade ≥3 treatment-related adverse events, and adverse events leading to treatment discontinuation.
